# Development of a dual fluorescent microsphere—based lateral flow immunoassays for simultaneous detection of *Brucella* and *Mycobacterium bovis* antibodies

**DOI:** 10.3389/fvets.2026.1881898

**Published:** 2026-08-11

**Authors:** Dihui Meng, Rongsheng Mi, Wei Zhu, Ying Zhang, Ke Lu, Zhaoguo Chen, Xiangan Han, Quan Wang, Jin Ni, Jun Wu, Baoping Guo, Lixin You, Guodong Mu

**Affiliations:** 1 College of Food Science and Engineering, Jilin Agricultural University, Changchun, China; 2 Key Laboratory of Animal Parasitology of Ministry of Agriculture, Laboratory of Quality and Safety Risk Assessment for Animal Products on Biohazards (Shanghai) of Ministry of Agriculture, Shanghai Veterinary Research Institute, Chinese Academy of Agricultural Sciences, Shanghai, China; 3 College of Life Sciences, Changchun Sci-Tech University, Changchun, China; 4 Tengzhou Animal Disease Prevention and Control Center of Shandong Province, Tengzhou, China; 5 Jinhua Key Laboratory of Genetic Breeding and Improvement on Jinhua Pig, College of Agriculture, Jinhua University of Vocational Technology, Jinhua, China; 6 State Key Laboratory of Pathogenesis, Prevention and Treatment of High Incidence Diseases in Central Asia, Xinjiang Medical University, and WHO-Collaborating Centre for Prevention and Care Management of Echinococcosis, The First Affiliated Hospital of Xinjiang Medical University, Urumqi, China; 7 Jilin Center for Animal Disease Control and Prevention, Changchun, China

**Keywords:** *Brucella* spp., immunochromatographic strip, lipopolysaccharide (LPS), MPB70, *Mycobacterium bovis*

## Abstract

**Introduction:**

Bovine brucellosis and bovine tuberculosis are important zoonotic diseases that pose serious threats to human and animal health and cause substantial economic losses in the livestock industry. Therefore, a rapid diagnostic method suitable for the simultaneous field detection of both diseases is urgently needed.

**Methods:**

Fluorescent microspheres conjugated with recombinant streptococcal protein G (rSPG) were prepared and used as tracers. *Brucella* lipopolysaccharide (LPS) and *Mycobacterium bovis* MPB70 protein were coated onto test lines 1 and 2 (TL1 and TL2), respectively, whereas rSPG was coated onto the control line (CL). This configuration enabled the development of a dual-antibody rapid test strip for the simultaneous detection of *Brucella* spp. and *M. bovis* antibodies. The optimal reaction conditions and performance characteristics of the test strip were systematically evaluated.

**Results and discussion:**

The dual rapid test strip enabled detection to be completed within 20 min. The optimal coating concentrations were determined to be 0.5 mg/mL for the Brucella test line (TL1) and 1.0 mg/mL for the *M. bovis* test line (TL2), and the optimal conjugation concentration of fluorescent microspheres was 0.25 mg/mL. Using a fluorescence reader, the minimum detection thresholds, expressed as the ratios of the fluorescence intensity of the test line to that of the control line (*T*/*C*), were determined to be 0.04109 for *Brucella* and 0.0276 for *M. bovis*. The dual test strip showed high specificity without detectable cross-reactivity, higher sensitivity than commercial colloidal gold test strips, and excellent intra- and inter-batch reproducibility (coefficients of variation <10%). Preliminary testing of 67 clinical samples showed that the dual test strip achieved agreement rates of 92.54% and 95.52% with commercial ELISA antibody detection kits for bovine brucellosis and bovine tuberculosis, respectively. A fluorescent microsphere-based dual-antibody rapid test strip was successfully developed for the simultaneous detection of antibodies against *Brucella* spp. and *M. bovis*. Exhibiting high sensitivity and specificity, this test strip serves as a highly suitable tool for the rapid diagnosis of bovine brucellosis and bovine tuberculosis antibodies.

## 1 Introduction

Bovine brucellosis and bovine tuberculosis are major zoonotic diseases that pose serious threats to livestock production and public health. Both diseases are listed as notifiable diseases by the World Organization for Animal Health (WOAH) (1, 2) and are classified as Category II animal diseases subject to priority control in China. Both *Brucella* spp. and *Mycobacterium bovis* can cause reproductive disorders, reduced productivity, and even death in cattle and sheep, resulting in substantial economic losses in the livestock industry (3). At present, prevention and control strategies for these two diseases in China are primarily based on an integrated approach combining immunization, continuous surveillance, and culling of positive animals. Among these measures, the establishment of rapid and accurate diagnostic methods is a critical prerequisite for effective surveillance and eradication programs.

*Brucella* spp. are small, Gram-negative, non-spore-forming, nonmotile coccobacilli that lack flagella and behave as facultative intracellular pathogens (4, 5). To date, 12 species have been reported within this genus, among which *Brucella abortus*, the primary causative agent of bovine brucellosis, is one of the most important species affecting cattle herds (6). This bacterium primarily invades the bovine reproductive system (7), leading to abortion and infertility in cows, orchitis and reduced breeding capacity in bulls, and death in calves (8, 9). Notably, some infected cattle may become asymptomatic carriers, shedding bacteria for prolonged periods without obvious clinical signs (10), thereby posing a serious challenge to disease screening and eradication efforts (11).

Bovine tuberculosis is primarily caused by *M. bovis* (12). This bacterium is a slender, slightly curved, acid-fast rod, whose cell wall contains lipids account for up to 60% of dry weight, which confers strong resistance to desiccation; however, it remains sensitive to moist heat and ethanol. Transmission occurs primarily through the respiratory and digestive tracts (13). Infected cattle typically experience a long incubation period and insidious clinical manifestations, and the bacterium can invade multiple organs, resulting in granulomatous necrosis (14, 15). Owing to its zoonotic potential and substantial impact on livestock productivity, this disease poses a serious threat to public health and the sustainable development of the livestock industry (16). Moreover, its complex epidemiological characteristics and slow disease progression further complicate prevention and control efforts (17).

Currently, diagnostic methods for *Brucella* spp. and *M. bovis* infections can be broadly classified into three categories: bacteriological, immunological, and molecular assays. Bacteriological methods rely on bacterial culture and identification; however, these procedures are time-consuming, require manipulation of viable organisms, pose substantial biosafety risks, and must be conducted in specialized laboratory facilities. Molecular assays, such as PCR, offer high sensitivity and specificity; however, sample pretreatment may still involve operator exposure to pathogenic organisms, and specialized instruments and technical expertise are required. Immunological methods, particularly serum antibody detection assays, are the most widely used diagnostic tools; however, existing serological assays have inherent limitations and do not fully meet the requirements of diverse screening scenarios (18). Specifically, the enzyme-linked immunosorbent assay (ELISA) offers high sensitivity and specificity and is suitable for large-scale batch testing; however, it requires specialized equipment, incurs relatively high costs, and can be affected by compromised sample quality, such as hemolysis (19). The complement fixation test (CFT) is highly specific and serves as an important confirmatory reference method; however, it is labor-intensive, requires stringent reaction conditions, and is difficult to implement for large-scale screening in field or primary-level settings. For bovine brucellosis, the Rose Bengal plate test (RBPT) is easy to perform and inexpensive, making it the preferred method for preliminary on-site screening (20); however, it has limited specificity, is prone to false-positive results, and cannot reliably distinguish vaccine-induced antibodies from infection-induced antibodies (21). The standard tube agglutination test (SAT) can be used to quantitatively measure antibody titers; however, the procedure is complex, and interpretation of results is subjective (22, 23). The indirect hemagglutination assay (IHA) does not require specialized equipment and is suitable for primary-level testing; however, its limited sensitivity may result in false-negative diagnoses (24). For bovine tuberculosis, the interferon-gamma release assay (IGRA) is widely used because it enables early detection and can effectively differentiate between vaccinated and naturally infected animals; however, it requires strict sample storage and transportation conditions and is costly, limiting its application in field or primary-level settings (25).

In summary, existing diagnostic technologies are predominantly laboratory-based and have substantial limitations in terms of testing efficiency, cost, and applicability in field or primary-level settings (26), highlighting the urgent need for rapid on-site diagnostic tools for *Brucella* spp. and *M. bovis* infections. At present, most rapid tests based on immunochromatography use colloidal gold as the labeling material. However, colloidal gold-based assays have several limitations, including relatively low sensitivity and poor inter-batch stability. In contrast, fluorescent microsphere-based immunochromatographic assays not only exhibit higher sensitivity but also use microspheres with uniform particle sizes and good inter-batch stability. Consequently, they have been widely applied in the diagnosis of various diseases. Furthermore, because most currently available diagnostic methods target a single pathogen; screening for both bovine brucellosis and bovine tuberculosis is time-consuming and labor-intensive. Therefore, a multiplex detection method capable of simultaneously detecting both pathogens is highly desirable. To address this gap, *Brucella* lipopolysaccharide (LPS) and *M. bovis* MPB70 protein were used as detection antigens in this study to develop a dual rapid test strip for the simultaneous detection of antibodies against *Brucella* spp. and *M. bovis*. This study aimed to provide a simple and user-friendly diagnostic tool for the rapid on-site screening of bovine brucellosis and bovine tuberculosis, thereby supporting field surveillance and eradication programs.

## 2 Materials and methods

### 2.1 Experimental materials

The recombinant protein rMPB70 was previously prepared in our laboratory. Briefly, the full-length gene was synthesized based on the coding sequence of *M. bovis* MPB70 available in GenBank (accession no. M33916) and cloned into the pET30a expression vector to generate the recombinant plasmid pET30a-MPB70. The recombinant protein rMPB70 was subsequently expressed following IPTG induction, purified, and used in the subsequent experiments. The recombinant streptococcal protein G (rSPG) was purchased from Luoyang Baisheng Biotechnology Co., Ltd. (Luoyang, China), and *Brucella* lipopolysaccharide (LPS) was purchased from Hangzhou Zhixian Biotechnology Co., Ltd. (Hangzhou, China). The positive and negative sera used in this study were obtained from dairy cattle serum samples previously collected by our laboratory from several provinces in China, including Ningxia, Hebei, and Jilin. The samples were screened and characterized using commercial ELISA kits of *Brucella* (Beijing Mingrida Technology Development Co., Ltd.) and *M. bovis* (Wuhan Keqian Biological Co., Ltd.), respectively. Positive sera containing antibodies against bovine viral diarrhea virus (BVDV), *Mycobacterium paratuberculosis*, bovine pathogenic *Escherichia coli*, and bovine herpesvirus type 1 (BoHV-1) were maintained by the Animal-Derived Food Safety Team at the Shanghai Veterinary Research Institute, Chinese Academy of Agricultural Sciences.

### 2.2 Reagents and materials

Fluorescent microspheres were purchased from Suzhou Weidu Biotechnology Co., Ltd. (Suzhou, China). Bovine serum albumin (BSA), Tween 20, PC-300 bacteriostatic preservative (ProClin 300), and polyvinylpyrrolidone (PVP) were purchased from Beijing Solarbio Science & Technology Co., Ltd. (Beijing, China). 2-(N-morpholino) ethanesulfonic acid (MES), 1-ethyl-3-(3-dimethylaminopropyl) carbodiimide hydrochloride (EDC), and N-hydroxysuccinimide (NHS) were purchased from Sigma-Aldrich (Shanghai) Trading Co., Ltd. (Shanghai, China). Materials used for test strip assembly, including nitrocellulose (NC) membranes, absorbent pads, and sample pads, were purchased from Shanghai Jiening Biotechnology Co., Ltd. (Shanghai, China). *Brucella* antibody colloidal gold test strips were purchased from Shenzhen Lvshiyuan Biotechnology Co., Ltd. (Shenzhen, China). *Mycobacterium bovis* MPB70/83 antibody detection test strips were purchased from Shenzhen Finder Biotechnology Co., Ltd. (Shenzhen, China). The sample diluent was prepared in purified water containing 7.0 g/L Na₂HPO₄·12H₂O, 0.446 g/L NaH₂PO₄·2H₂O, 10.0 g/L bovine serum albumin (BSA), 0.5 mL/L Tween 20, and 0.5 mL/L ProClin 300. The solution was mixed thoroughly and stored at 4 °C until use.

### 2.3 Preparation of fluorescent microsphere–rSPG conjugates

A 200-μL aliquot of fluorescent microspheres was transferred to a 1.5-mL centrifuge tube. NHS (50 μL, 10 mg/mL) and EDC (50 μL, 20 mg/mL) were then added sequentially, and the suspension was gently shaken for 15 min to activate the microspheres. Subsequently, 400 μL of MES buffer (50 mM) was added, and activation was continued for an additional 15 min. After activation, the suspension was sonicated at 4% power for 10 min and centrifuged at 12,000 rpm for 20 min. The supernatant was discarded, and the pellet was resuspended in 200 μL of borate buffer (50 mM), followed by sonication using the same settings. Next, 100 μg of rSPG was added, and the final volume was adjusted to 300 μL with borate buffer. The mixture was gently mixed on a shaker at 4 °C for 24 h to facilitate conjugation. After conjugation, the mixture was sonicated using the same settings and centrifuged at 12,000 rpm for 10 min. The supernatant was discarded, and the pellet was resuspended in 500 μL of blocking buffer containing 5% BSA and incubated on a shaker at 4 °C for 6 h. After blocking, the sample was sonicated again and centrifuged at 12,000 rpm for 10 min. The supernatant was discarded, and the pellet was finally resuspended in 300 μL of dilution buffer. The microspheres were uniformly dispersed by sonication, and the resulting conjugated microspheres were stored at 4 °C in the dark.

### 2.4 Test strip assembly

**Figure 1:**
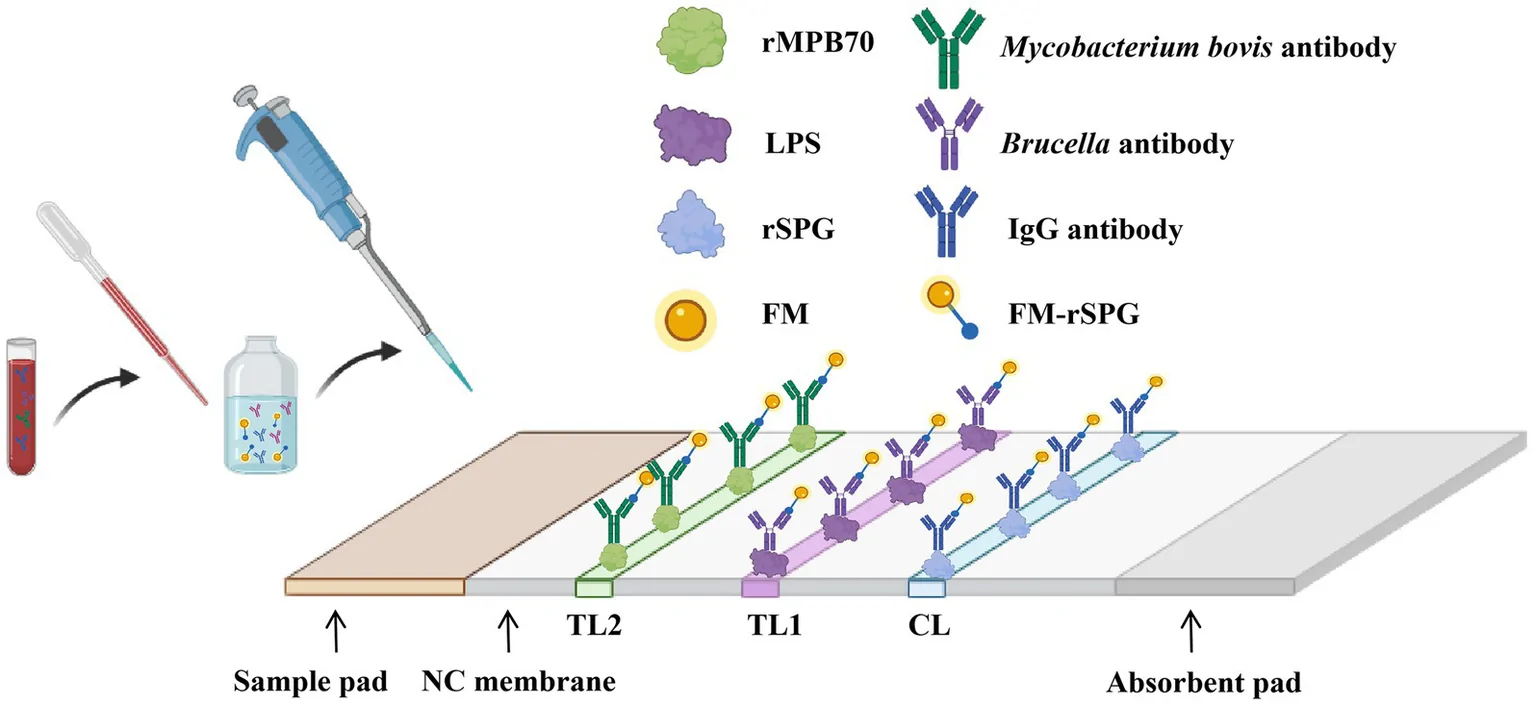
Schematic illustration of the assembly and working principle of the immunochromatographic test strip. During the assay, the serum sample was mixed with the FM-rSPG conjugate, and the resulting mixture migrates along the strip via capillary action. Antibodies in the sample were subsequently captured by *Brucella* LPS at test line 1 (TL1) or *Mycobacterium bovis* rMPB70 at test line 2 (TL2). The rSPG immobilized at the control line (CL) serves as a procedural control.

The glass fiber pad was immersed in the sample treatment solution for 5 min, then removed and dried in an oven at 37 °C. The dried glass fiber pad was sealed and stored for subsequent use as the sample pad. The nitrocellulose (NC) membrane was mounted onto the center of a polyvinyl chloride (PVC) backing card. Using a dispenser, *Brucella* LPS, *M. bovis* rMPB70, and rSPG were dispensed onto the NC membrane at 1 μL/cm to generate test line 1 (TL1), test line 2 (TL2), and the control line (CL), respectively. The distance between adjacent lines was maintained at 3 mm. After dispensing, the sample pad and absorbent pad were attached to opposite ends of the NC membrane, with each pad overlapping the NC membrane by 2 mm. The assembled PVC card was dried in an oven at 37 °C for 2 h and then cut into 3-mm-wide test strips using a strip cutter. Finally, the cut test strips were placed into plastic cassettes, sealed in aluminum foil bags containing desiccant, vacuum-packed, and stored at 4 °C until use (Figure 1).

The serum sample was mixed with the FM-rSPG conjugate, and the mixture was applied to the sample pad. Driven by capillary action, the mixture migrated along the test strip. When the mixture reached the test lines, the target antibodies, if present in the serum sample, bound to the corresponding immobilized antigens, forming either FM-rSPG–anti-*Brucella* antibody–LPS complexes at TL1 or FM-rSPG–anti-*M. bovis* antibody–rMPB70 complexes at TL2, thereby producing detectable signals at the corresponding test lines. The unbound components continued to migrate toward the control line, where FM-rSPG–IgG–rSPG complexes were formed, producing a signal at the CL. If the serum sample did not contain either target antibody, no antigen–antibody complexes were formed at TL1 or TL2, and thus no signals were observed at the test lines. In this case, a signal was produced only at the CL, indicating a negative result.

### 2.5 Interpretation of test strip results

**Figure 2:**
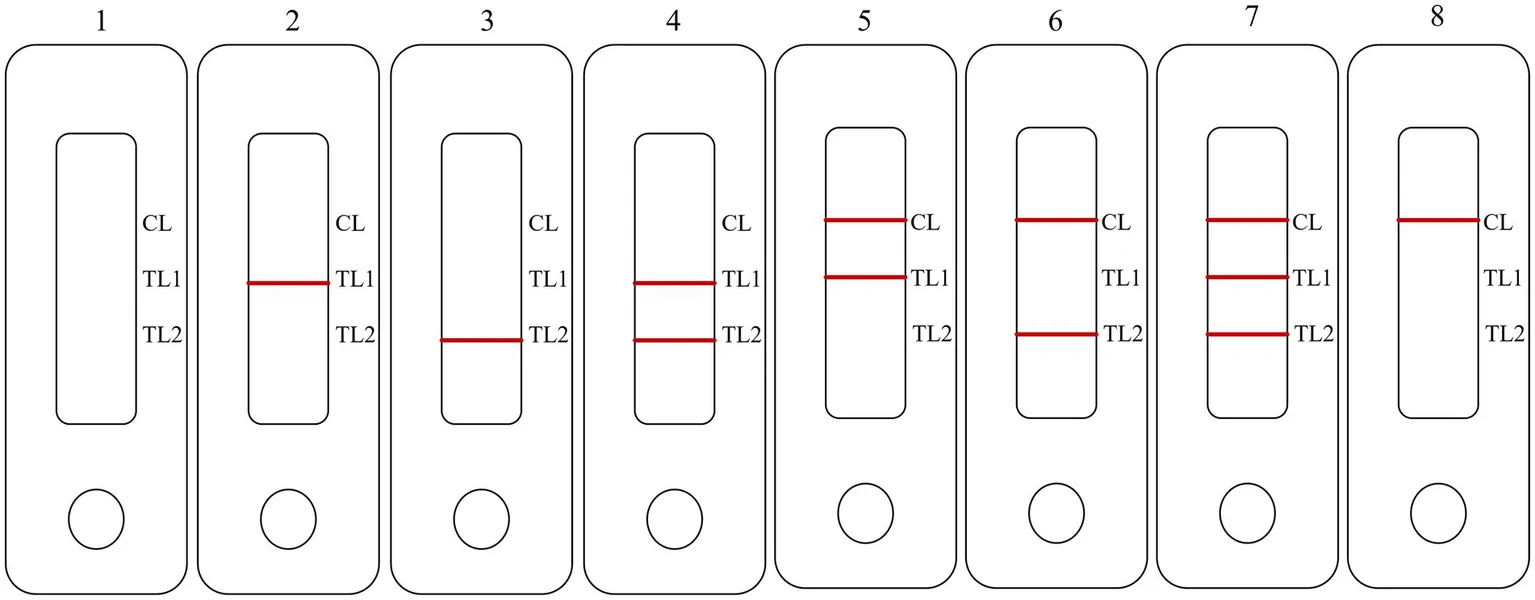
Schematic illustration of the interpretation criteria for the fluorescent microsphere-based test strip. Strips 1–4 indicate invalid results; Strip 5 indicates seropositivity for *Brucella* spp.; Strip 6 indicates seropositivity for *Mycobacterium bovis*; Strip 7 indicates seropositivity for both *Brucella* spp. and *Mycobacterium bovis*; and Strip 8 indicates seronegativity for both *Brucella* spp. and *Mycobacterium bovis*.

For detection, 4 μL of serum was mixed with 100 μL of sample diluent and incubated in the dark for 5 min. Subsequently, 60–100 μL of the mixture was applied to the sample well. After a 10-min reaction, the bands were observed under an ultraviolet (UV) lamp, or fluorescence signals were measured using a fluorescence reader (Suzhou Hemai Precision Instruments Co., Ltd., Suzhou, China). If no fluorescent band appeared on the control line (CL), the result was considered invalid. If a fluorescent band appeared on the CL, the test was considered valid. For valid tests, the results were interpreted as follows: fluorescent bands on both the CL and test line 1 (TL1) indicated positivity for antibodies against *Brucella* spp.; fluorescent bands on both the CL and test line 2 (TL2) indicated positivity for antibodies against *M. bovis*; simultaneous fluorescent bands on the CL, TL1, and TL2 indicated positivity for antibodies against both *Brucella* spp. and *M. bovis*; and a fluorescent band only on the CL indicated a negative result (Figure 2).

### 2.6 Optimization of antigen coating concentrations on the test lines

Different concentrations of LPS and rMPB70 (1.0, 0.5, and 0.25 mg/mL) were applied to test line 1 (TL1) and test line 2 (TL2), respectively. The fluorescence intensity of each test line was measured using a fluorescence reader to determine the optimal antigen coating concentrations. The *Brucella* positive and *M. bovis* positive sera were screened in our laboratory by ELISA kits; during use, they were diluted 1:100 as the source of target antibodies for optimizing other test-strip conditions.

### 2.7 Optimization of coupling conditions between fluorescent microspheres and rSPG

Different concentrations of rSPG (0.125, 0.25, and 0.5 mg/mL) were conjugated to 200 μL of fluorescent microspheres according to the coupling procedure described in Section 2.3, with only the rSPG concentration varied. The fluorescence intensity obtained at each rSPG concentration was measured using a fluorescence reader to determine the optimal rSPG conjugation concentration.

### 2.8 Determination of cutoff values for the test strip

Twenty-five serum samples from healthy cattle that were negative for antibodies against both *Brucella* spp. and *M. bovis* were tested under the optimized reaction conditions. Fluorescent bands were first observed under a UV lamp, and the fluorescence signal intensities of the test line (*T* value) and control line (*C* value) for each sample were then measured using a fluorescence reader. The ratio of the fluorescence intensity at the test line to that at the control line (*T*/*C* value) was used to determine the detection result of each sample. Based on the *T*/*C* ratios of the negative samples, the cutoff value was determined using the following formula: cutoff value = mean *T*/*C* ratio of negative samples + 3 × standard deviation (SD). A sample was considered positive when its *T*/*C* ratio was equal to or greater than the cutoff value; otherwise, it was considered negative.

### 2.9 Analytical sensitivity of the test strip

Bovine sera positive for antibodies against *Brucella* spp. and *M. bovis*, with an endpoint titer of 1,600 as determined using an indirect ELISA kit, were mixed at equal volumes. The pooled serum was serially diluted to 1:100, 1:200, 1:400, 1:800, and 1:1,600 and tested using both the fluorescent microsphere-based test strip developed in this study and a commercial colloidal gold test strip to evaluate analytical sensitivity. Each dilution was tested in triplicate.

### 2.10 Analytical specificity of the test strip

To evaluate the analytical specificity of the fluorescent microsphere-based test strip, sera positive for antibodies against various pathogens were tested, including BVDV, *M. paratuberculosis*, bovine pathogenic *E. coli*, and BoHV-1.

### 2.11 Repeatability of the test strip

Four *Brucella*-positive sera and four *M. bovis*-positive sera, as confirmed by commercial ELISA kits, were separately selected. Each serum sample was diluted 1:200, and the diluted sera were then mixed at equal volumes for the repeatability experiments. These samples were used for intra-batch and inter-batch repeatability analyses. For intra-batch repeatability, each of the four serum samples was tested five times under identical conditions using test strips from the same batch, and the intra-batch coefficient of variation (CV) was calculated. For inter-batch repeatability, test strips from three different batches were used to test the same four serum samples in triplicate, and the inter-batch CV was calculated.

### 2.12 Agreement analysis of the test strip

A total of 67 serum samples were randomly collected from a dairy farm in Jilin Province, China, and tested in parallel using the fluorescent microsphere-based test strip developed in this study and commercial indirect ELISA antibody detection kits for bovine brucellosis and bovine tuberculosis. Using the ELISA results as the reference standard, the relative sensitivity, relative specificity, and overall agreement rate of the test strip were calculated to evaluate its diagnostic performance. The overall agreement rate was defined as the number of samples with concordant results between the two methods divided by the total number of samples × 100%; relative sensitivity was defined as the number of test strip-positive samples among ELISA-positive samples divided by the total number of ELISA-positive samples × 100%; and relative specificity was defined as the number of test strip-negative samples among ELISA-negative samples divided by the total number of ELISA-negative samples × 100%. In addition, Cohen’s kappa coefficient was used to assess agreement between the two methods.

## 3 Results

### 3.1 Assembly of the test strip

**Figure 3:**
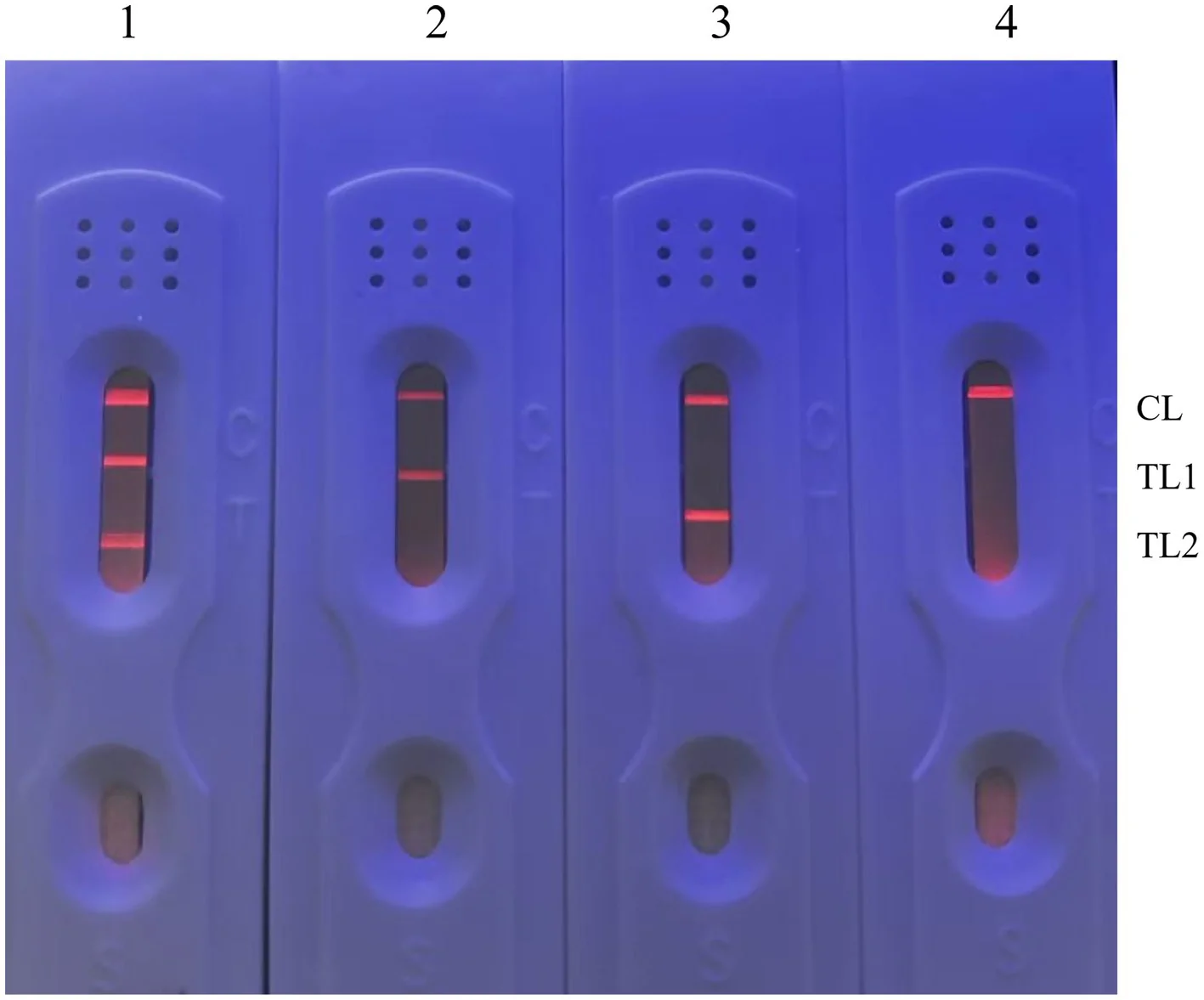
Visualization of test strip results under ultraviolet light. Strip 1 indicates seropositivity for both *Brucella* spp. and *Mycobacterium bovis*; strip 2 indicates seropositivity for *Brucella* spp.; strip 3 indicates seropositivity for *Mycobacterium bovis*; and strip 4 indicates seronegativity.

The sample pad was loaded with fluorescent microspheres conjugated to rSPG. Test line 1 (TL1) was coated with *Brucella* LPS, test line 2 (TL2) with *M. bovis* rMPB70 protein, and the control line (CL) with rSPG. Based on this design, a fluorescent microsphere-based immunochromatographic test strip was successfully constructed. The strip was able to detect antibodies against *Brucella* spp. and *M. bovis* and to identify cattle serum samples positive for antibodies against both pathogens. Positive results were visualized as distinct fluorescent bands under 365-nm UV irradiation (Figure 3).

### 3.2 Detection results for test lines coated with different antigen concentrations

**Figure 4:**
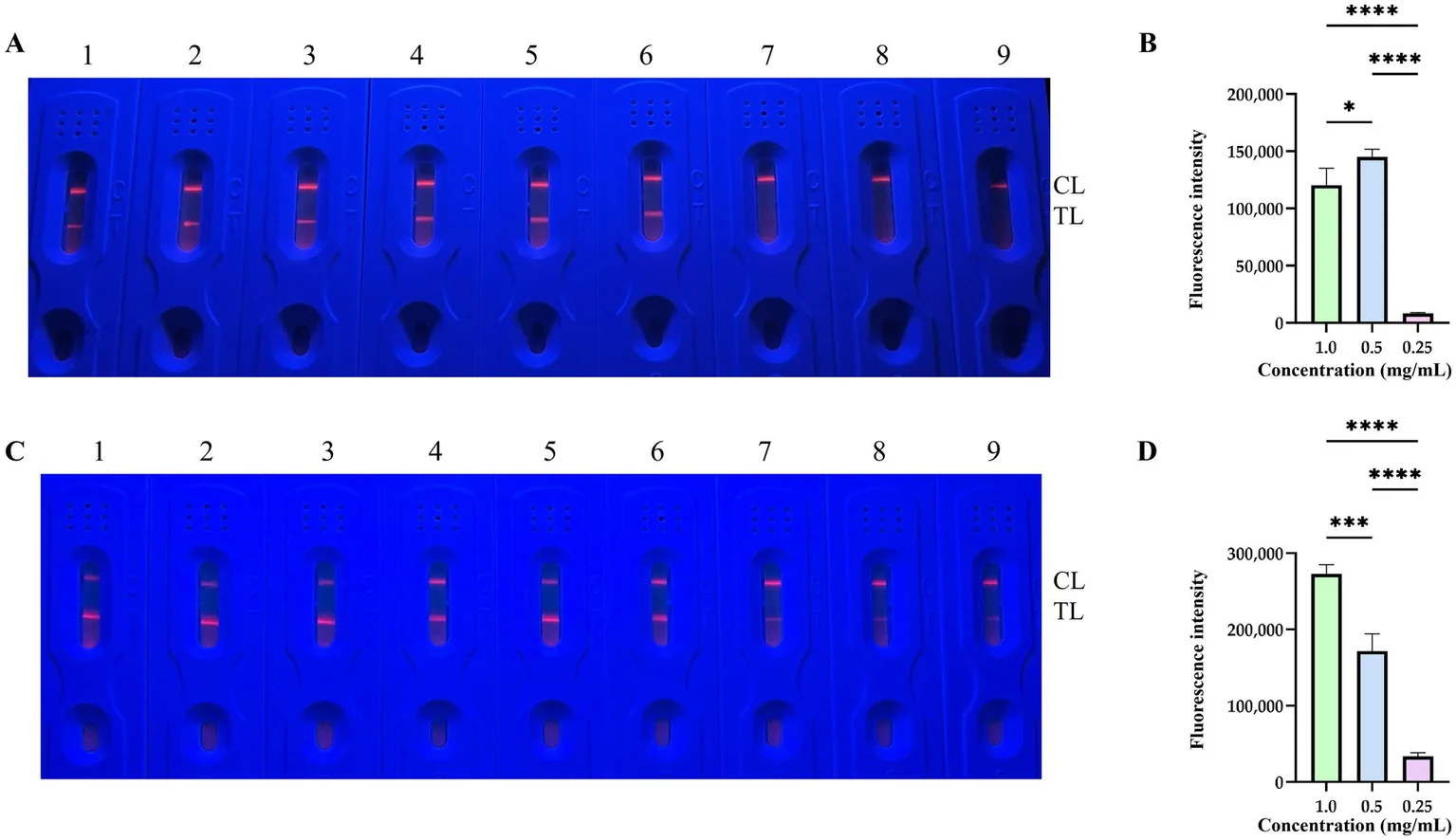
Detection performance of test lines coated with different antigen concentrations. **(A)** Optimization of the LPS coating concentration on TL1 under UV observation. **(B)** Quantitative fluorescence analysis of TL1 coated with different LPS concentrations. **(C)** Optimization of the rMPB70 coating concentration on TL2 under UV observation. **(D)** Quantitative fluorescence analysis of TL2 coated with different rMPB70 concentrations. In panels **(A–C)**, strips 1–3, 4–6, and 7–9 correspond to antigen concentrations of 1.0, 0.5, and 0.25 mg/mL, respectively. Asterisks indicate statistically significant differences between groups (*, *p* < 0.05; ***, *p* < 0.001; and ****, *p* < 0.0001).

To determine the optimal antigen coating concentrations, TL1 and TL2 were coated with different concentrations of LPS and rMPB70, respectively. Clear fluorescent bands were visible under UV light when both LPS and rMPB70 were coated at concentrations of 1.0 and 0.5 mg/mL. However, when the LPS coating concentration was reduced to 0.25 mg/mL, no fluorescent band was observed, whereas a weak fluorescent band remained visible for rMPB70 at the same concentration (Figures 4A,C). Quantitative fluorescence analysis further showed that, for LPS, the highest fluorescence intensity was observed at 0.5 mg/mL (145,071), followed by 1.0 mg/mL (120,177), with a significant difference between the two concentrations (*p* < 0.05). The lowest fluorescence intensity was recorded at 0.25 mg/mL (8,382), which was significantly lower than those observed at 0.5 and 1.0 mg/mL (*p* < 0.0001; Figure 4B). In contrast, the fluorescence intensity of rMPB70 decreased progressively as the coating concentration decreased, with values of 272,915, 171,557, and 33,472 at 1.0, 0.5, and 0.25 mg/mL, respectively. Pairwise comparisons among the three concentrations showed significant differences (*p* < 0.0001; Figure 4D). Therefore, 0.5 mg/mL LPS and 1.0 mg/mL rMPB70 were selected as the optimal coating concentrations for the test lines and used in all subsequent experiments.

### 3.3 Detection results for microspheres coupled with different concentrations of rSPG protein

**Figure 5:**
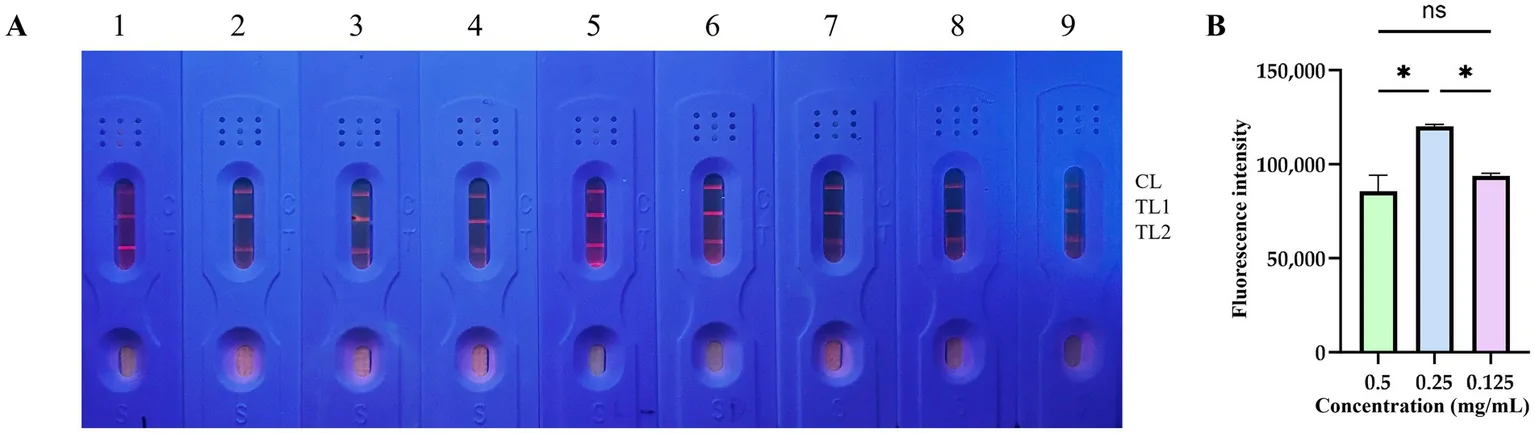
Optimization of rSPG conjugation to fluorescent microspheres. **(A)** UV visualization of fluorescent microsphere-rSPG conjugates prepared with different rSPG concentrations. **(B)** Quantitative fluorescence analysis of fluorescent microsphere-rSPG conjugates prepared with different rSPG concentrations. Strips 1–3, 0.5 mg/mL rSPG; strips 4–6, 0.25 mg/mL rSPG; and strips 7–9, 0.125 mg/mL rSPG.

Different concentrations of rSPG were conjugated to fluorescent microspheres. Clear fluorescent bands were visible under UV light at all three rSPG concentrations (Figure 5A). Quantitative analysis using a fluorescence analyzer showed that the fluorescence intensity was highest at an rSPG conjugation concentration of 0.25 mg/mL (115,000), followed by 0.125 mg/mL (95,000) and 0.5 mg/mL (85,508). The fluorescence intensity in the 0.25 mg/mL group was significantly higher than those in the 0.5 and 0.125 mg/mL groups (*p* < 0.05; Figure 5B). Therefore, 0.25 mg/mL rSPG was selected as the optimal concentration for conjugation with fluorescent microspheres and used in all subsequent experiments.

### 3.4 Determination of cutoff values for the test strip

To determine the cutoff values for the fluorescent microsphere-based immunochromatographic test strip, 25 serum samples negative for antibodies against both *Brucella* spp. and *M. bovis* were quantitatively analyzed using a fluorescence reader. For *Brucella* spp., the mean *T*/*C* value was 0.00986, the standard deviation (SD) was 0.01041, and the corresponding cutoff value was 0.04109. For *M. bovis*, the mean *T*/*C* value was 0.00424, the SD was 0.00781, and the corresponding cutoff value was 0.02768 (Table 1).

**Table 1:** *T*/*C* values and cutoff values of the test strip for negative serum samples.

Serum samples	*T/C* values of serum samples									
	*Brucella*					*Mycobacterium bovis*				
1–5	0.01398	0.00930	0.00645	0.00415	0.00497	0.00000	0.00307	0.03799	0.01336	0.00000
6–10	0.00842	0.00566	0.00746	0.00764	0.00667	0.00557	0.00431	0.00000	0.00000	0.00239
11–15	0.00198	0.00784	0.00930	0.01073	0.01844	0.00241	0.00000	0.00225	0.00000	0.00000
16–20	0.05622	0.00536	0.01367	0.00279	0.00666	0.00190	0.00000	0.00370	0.00000	0.00000
21–25	0.01260	0.00130	0.00748	0.00935	0.00796	0.00273	0.00999	0.00634	0.00615	0.00378
Mean	0.00986					0.00424				
SD	0.01041					0.00781				
Cut-off	0.04109					0.02768				

### 3.5 Analytical sensitivity of the test strip

**Figure 6:**
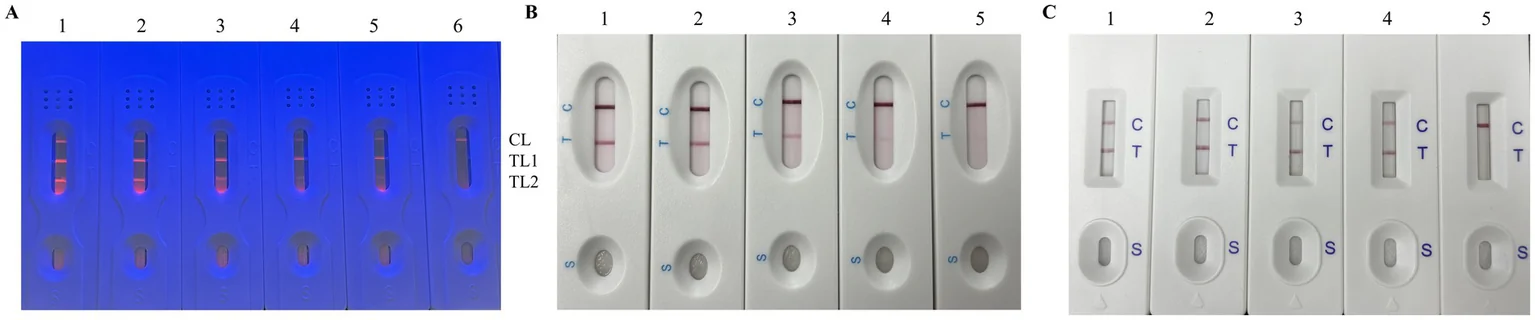
Analytical sensitivity of the test strips. **(A)** Analytical sensitivity of the fluorescent microsphere-based immunochromatographic test strip developed in this study at various serum dilutions. **(B)** Analytical sensitivity of a commercial *Brucella* lateral flow test strip. **(C)** Analytical sensitivity of a commercial *Mycobacterium bovis* lateral flow test strip. In panels **(A–C)**, strip 1–6 correspond to serum dilutions of 1:50, 1:100, 1:200, 1:400, 1:800, and 1:1,600, respectively.

Serum samples positive for antibodies against *Brucella* spp. and *M. bovis* were serially diluted and tested using both the fluorescent microsphere-based immunochromatographic test strip developed in this study and a commercial colloidal gold strip to evaluate analytical sensitivity. The results are shown in Figure 6A. Clear fluorescent bands were still visible under a UV lamp when the positive sera were diluted to 1:400. At a dilution of 1:800, a distinct fluorescent band was observed on the *Brucella* test line (TL1), whereas the band on the *M. bovis* test line (TL2) was relatively weak. At a dilution of 1:1,600, no fluorescent bands were detected on either TL1 or TL2. Quantitative analysis using a fluorescence reader further confirmed that, at a 1:800 dilution, the samples remained positive for antibodies against both *Brucella* spp. and *M. bovis*, whereas at a 1:1,600 dilution, both were negative (Table 2). The same set of diluted sera was tested using a commercial colloidal gold strip. At a dilution of 1:800, no visible bands were observed on the test lines for either *Brucella* spp. or *M. bovis*, and the results were therefore interpreted as negative (Figures 6B,C). These findings demonstrate that the analytical sensitivity of the fluorescent microsphere-based immunochromatographic test strip was superior to that of the commercial colloidal gold strip.

**Table 2:** Comparative analytical sensitivity of the fluorescent microsphere-based immunochromatographic test strip and commercial colloidal gold strips.

Serum dilution	Fluorescent microsphere-based test strip		Commercial colloidal gold test strip	
	*Brucella* (*T*/*C*)	*Mycobacterium bovis* (*T*/*C*)	*Brucella*	*Mycobacterium bovis*
1:200	+(7.797)	+(5.503)	+	+
1:400	+(6.585)	+(3.022)	+	+
1:800	+(4.379)	+(0.637)	−	−
1:1,600	−(0.021)	−(0.000)	−	−

“+” indicates a seropositive sample; “−” indicates a seronegative sample. Values in parentheses represent the *T*/*C* ratios measured using the fluorescence reader.

### 3.6 Analytical specificity of the test strip

**Figure 7:**
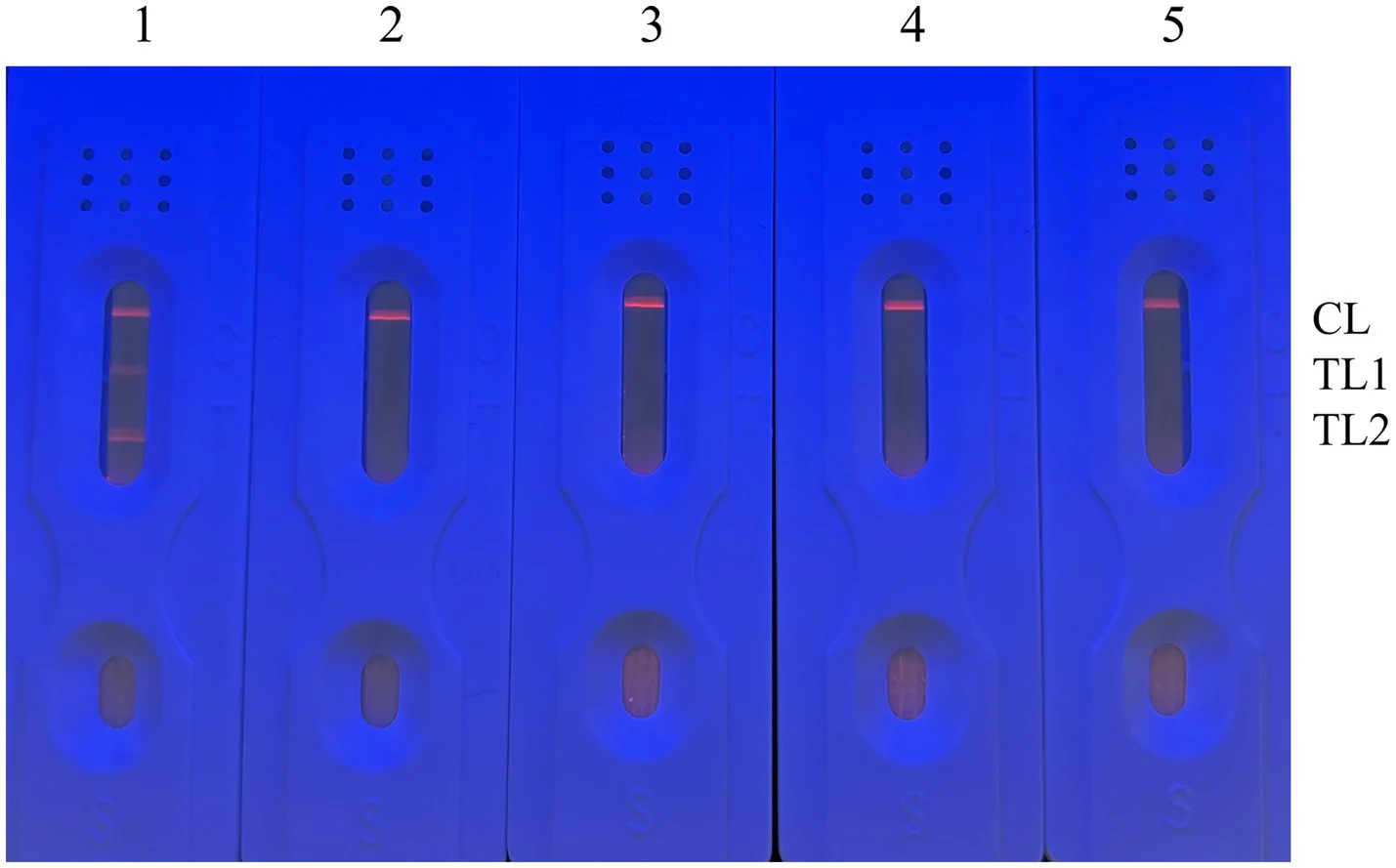
Analytical specificity of the fluorescent microsphere-based immunochromatographic test strip under UV light. Strip 1 indicates seropositivity for both *Brucella* spp. and *Mycobacterium bovis*; strip 2 indicates seropositivity for BVDV; strip 3 indicates seropositivity for *Mycobacterium paratuberculosis*; strip 4 indicates seropositivity for bovine pathogenic *Escherichia coli*; and strip 5 indicates seropositivity for BoHV-1.

To evaluate the analytical specificity of the developed test strip, sera positive for antibodies against four different microorganisms were tested. The results are shown in Figure 7. Under UV light, fluorescent bands were observed only for sera positive for antibodies against *Brucella* spp. and *M. bovis*, whereas all sera positive for antibodies against other bovine pathogens tested negative. These findings indicate that the test strip exhibited high analytical specificity with no detectable cross-reactivity with sera positive for antibodies against other pathogens.

### 3.7 Repeatability of the test strip

Intra-batch and inter-batch repeatability were evaluated using four serum samples positive for antibodies against both *Brucella* spp. and *M. bovis*. Each sample was tested five times using test strips from the same batch for intra-batch repeatability and in triplicate using test strips from three different batches for inter-batch repeatability. For *Brucella* spp., the intra-assay coefficients of variation (CVs) ranged from 5.2 to 7.5%, and the inter-assay CVs ranged from 7.3 to 9.2%. For *M. bovis*, the intra-assay CVs ranged from 5.1 to 7.8%, and the inter-assay CVs ranged from 7.9 to 9.4% (Table 3). All intra-assay and inter-assay CVs for both targets were below 10%, indicating that the fluorescent microsphere-based immunochromatographic test strip developed in this study exhibited satisfactory repeatability.

**Table 3:** Repeatability of the fluorescent microsphere-based immunochromatographic test strip.

Pathogen	Sample	Intra-assay CV (%)	Inter-assay CV (%)
*Brucella*	M1	7.5	9.2
	M2	5.2	7.3
	M3	6.7	7.5
	M4	6.3	8.7
*Mycobacterium bovis*	M1	7.8	9.4
	M2	6.5	8.1
	M3	7.4	8.8
	M4	5.1	7.9

### 3.8 Agreement analysis of the test strip

A total of 67 bovine serum samples randomly collected from cattle farms were tested using both the fluorescent microsphere-based immunochromatographic test strip developed in this study and commercial ELISA kits to assess agreement in the detection of antibodies against *Brucella* spp. and *M. bovis*. For *Brucella* spp., the test strip identified 21 antibody-positive samples, whereas ELISA identified 22, with 19 samples testing positive by both methods; this yielded a positive percent agreement (PPA) of 86.36% (19/22). For negative results, the test strip identified 46 antibody-negative samples, whereas ELISA identified 45, with 43 concordant negative results, yielding a negative percent agreement (NPA) of 95.56% (43/45). The overall percent agreement (OPA) was 92.54% (62/67), with a Cohen’s kappa coefficient of 0.83 (Tables 4, 5). For *M. bovis*, the test strip identified six antibody-positive samples, whereas ELISA identified five, with four samples testing positive by both methods (PPA = 80.00%, 4/5). The test strip yielded 61 antibody-negative results, whereas ELISA yielded 62, with 60 concordant negative results (NPA = 96.77%, 60/62). The OPA was 95.52% (64/67), with a Cohen’s kappa coefficient of 0.70 (Tables 4, 5). According to standard kappa interpretation criteria, the coefficient of 0.83 for *Brucella* spp. indicated almost perfect agreement, whereas the coefficient of 0.70 for *M. bovis* indicated substantial agreement. Overall, the developed fluorescent microsphere-based immunochromatographic test strip demonstrated good agreement with commercial ELISA kits and may serve as a suitable tool for rapid on-site testing.

**Table 4:** Agreement between the immunochromatographic test strip and commercial ELISA kits.

Fluorescent microsphere strip test	Commercial ELISA kit for *Brucella*			Commercial ELISA kit for *Mycobacterium bovis*		
	Positive	Negative	Total	Positive	Negative	Total
Positive	19	2	21	4	2	6
Negative	3	43	46	1	60	61
Total	22	45	67	5	62	67

**Table 5:** Agreement analysis between the immunochromatographic test strip and ELISA kits.

Evaluation metrics	*Brucella*	*Mycobacterium bovis*
Positive percent agreement	86.36%	80.00%
Negative percent agreement	95.56%	96.77%
Overall percent agreement	92.54%	95.52%
*κ* value	0.83	0.7

## 4 Discussion

Brucellosis and bovine tuberculosis are important zoonotic diseases that continue to threaten the global livestock industry and public health. Brucellosis can cause reproductive disorders in livestock and may be transmitted to humans, leading to clinical manifestations such as undulant fever. Bovine tuberculosis, primarily caused by *M. bovis*, results in substantial economic losses to the livestock industry and poses a risk of cross-species transmission. The prevention and control of these two diseases have long relied on diagnostic approaches such as the Rose Bengal test (RBT) and enzyme-linked immunosorbent assay (ELISA) for brucellosis, as well as the single intradermal comparative cervical tuberculin test (SICCT) and interferon-gamma release assay (IGRA) for bovine tuberculosis. However, these methods have several limitations that hinder effective disease surveillance and eradication programs. Islam et al. (27) reported that the detection rate of *Brucella* in serologically negative animals reached 35.08%, and similar false-negative issues have been reported for SICCT and IGRA in bovine tuberculosis diagnosis, potentially compromising the implementation of test-and-slaughter strategies. Therefore, rapid, sensitive, and specific point-of-care testing methods are urgently needed for field surveillance and control programs.

In recent years, lateral flow immunochromatographic assays (LFIAs) have attracted increasing attention because of their simplicity, rapid turnaround time, and suitability for field use. Freddi et al. (28) established an LFIA-based method for brucellosis detection and compared it with WOAH-recommended RBT, CFT, and iELISA, demonstrating relative sensitivity and specificity above 97.25%, which supports the reliability of LFIA for on-site screening. Alonso et al. (29) developed a colloidal gold test strip for bovine tuberculosis detection with high specificity, which could help compensate for the limitations of the tuberculin skin test; however, its sensitivity remained inadequate, detecting only 51.72% (45/87) of ELISA-confirmed positive samples. Wang et al. (30) developed a fluorescent platform for detecting *Brucella* antibodies with high sensitivity, achieving a detection limit as low as 0.48 IU/mL and enabling rapid quantitative detection. However, that method targeted only a single pathogen and therefore could not meet the need for simultaneous multi-pathogen detection. Compared with these previous studies, the dual fluorescent microsphere-based test strip developed in this study enables simultaneous screening for antibodies against both *Brucella* spp. and *M. bovis* in a single assay. The entire detection process can be completed within 15 min, reducing testing time and cost while improving screening efficiency, making the method well suited for rapid field screening and primary-level disease surveillance.

In China, prevention and control strategies for brucellosis and bovine tuberculosis differ substantially, which directly affects the applicability of different diagnostic methods. For brucellosis control, local authorities are required to formulate immunization strategies according to regional epidemiological conditions. Large-scale farms generally implement immunization with live attenuated vaccines, making it difficult to distinguish vaccine-induced antibodies from those elicited by natural infection. This may limit the application of the *Brucella* antibody detection component of the method developed in this study in vaccinated herds. However, in remote areas of western China dominated by grazing or mixed crop–livestock systems, as well as in some backyard herds with limited awareness of disease prevention, vaccination is often not routinely implemented. Therefore, the rapid diagnostic method established in this study may have broad application prospects in non-vaccinated areas. For bovine tuberculosis, no ideal commercial vaccine is currently available for routine use in cattle. The existing bacillus Calmette–Guérin (BCG) vaccine shows unstable protective efficacy in cattle and interferes with the interpretation of routine diagnostic tests. Thus, regular surveillance and culling of positive animals remain the core strategies for bovine tuberculosis control in China, making rapid and accurate field diagnostic methods particularly important for improving surveillance efficiency. Overall, the dual rapid detection method for *Brucella* spp. and *M. bovis* established in this study holds promising application potential for brucellosis screening in non-vaccinated areas and routine bovine tuberculosis surveillance.

Compared with commercial single-plex test strips, the dual test strip developed in this study demonstrated higher analytical sensitivity. When sera positive for antibodies against *Brucella* spp. and *M. bovis* were diluted to 1:800, the test strip remained positive under ultraviolet excitation, whereas no visible test-line signal was observed for the commercial test strips at the same dilution. In the agreement analysis, using commercial ELISA kits as reference methods, the overall percent agreements of this test strip for *Brucella* spp. and *M. bovis* were 92.54 and 95.52%, with Cohen’s kappa coefficients of 0.83 and 0.70, respectively. These results indicate that the dual test strip showed substantial to almost perfect agreement with commercial ELISA kits. Therefore, the dual rapid detection method developed in this study balances diagnostic performance with field applicability. It simplifies laboratory-based ELISA testing into a one-step point-of-care testing (POCT) format, streamlines the operational workflow, and may be suitable for large-scale primary-level screening and epidemiological surveys. However, this study has certain limitations in comparative validation. Numerous commercial diagnostic test strips and ELISA kits are currently available, and their performance may vary across brands and formats. Due to cost constraints, only one commercial product of each type was selected for comparative analysis in this study, which limits the representativeness of the comparison. Future systematic comparative studies incorporating a broader range of commercial test strips and ELISA kits are needed to further validate the performance advantages of the developed test strip.

Currently, most rapid immunochromatographic detection methods use colloidal gold as the primary labeling material. For example, Zhu et al. (31) developed a colloidal gold diagnostic test strip targeting LPS antigen for the detection of human brucellosis, and Alonso et al. (29) developed a colloidal gold diagnostic test strip targeting MPB83 antigen for the detection of bovine tuberculosis. Although colloidal gold labeling technology offers advantages such as simple operation, low cost, and intuitive result interpretation, making it suitable for qualitative field screening, it also has notable limitations (32). First, its relatively low analytical sensitivity may lead to false-negative results. Second, batch-to-batch reproducibility may be poor because the stability of colloidal gold probes can vary across preparations or operators. Third, colloidal gold-based assays generally provide only qualitative or semi-quantitative results, making it difficult to meet the requirements of precise quantitative detection. In recent years, fluorescent microsphere-based immunochromatographic assays have been increasingly applied in the POCT field. Fluorescent microspheres are characterized by uniform particle size, strong fluorescence intensity, and good signal stability. Their sensitivity is generally higher than that of colloidal gold, enabling the effective detection of low-abundance antibodies in animals with subclinical or latent infections. Moreover, their batch-to-batch stability is superior to that of colloidal gold labeling. For instance, Zhang et al. (33) used fluorescent microspheres to label monoclonal antibodies against T-2 toxin and developed a rapid diagnostic test strip that outperformed a colloidal gold-based test strip. However, results obtained using fluorescent microsphere labeling cannot typically be interpreted directly by the naked eye and require observation under ultraviolet excitation light. This limitation can be addressed by using portable UV light sources or fluorescence readers. Portable UV flashlights are inexpensive and convenient for field interpretation, whereas dedicated fluorescence readers enable quantitative analysis based on fluorescence signal intensity. Thus, fluorescent microsphere labeling can support both qualitative detection and quantitative analysis, representing a functional advantage over colloidal gold labeling. In the future, the integration of mobile phone-based image recognition systems or portable fluorescence readers may further enhance the field applicability of fluorescent microsphere-based immunochromatographic technology. Overall, the dual diagnostic method developed in this study offers notable advantages in sensitivity, stability, and quantitative detection capability. Nevertheless, a colloidal gold-based rapid diagnostic method targeting the same analytes was not developed in this study, and only comparisons with commercial diagnostic test strips were performed. Future studies should develop a colloidal gold immunochromatographic assay targeting the same diagnostic antigens to enable a more direct comparison with the fluorescent microsphere-based method described herein.

The selection of diagnostic antigens is crucial for optimizing the performance of immunochromatographic assays. In this study, *Brucella* LPS and *M. bovis* MPB70 protein were selected as diagnostic antigens to develop a dual fluorescent microsphere-based POCT (point-of-care testing) method. As a major immunodominant antigen of *Brucella*, LPS can induce early, high-titer, and persistent antibody responses after infection, making it a widely recognized target for serological diagnosis of brucellosis. Cai et al. (34) established an indirect ELISA for brucellosis using LPS as the coating antigen, confirming the high sensitivity and reliability of LPS for serological detection. MPB70 is one of the major secreted proteins of *M. bovis* and exhibits high species specificity. Marassi et al. (35) established an ELISA method using MPB70 that could effectively distinguish bovine tuberculosis from bovine paratuberculosis. Miura et al. (36) further confirmed that MPB70 is expressed only in *M. bovis* and closely related strains possessing both high immunogenicity and high specificity. Therefore, the use of *Brucella* LPS and *M. bovis* rMPB70 in the present study provides a rational antigen combination for simultaneous serological screening of brucellosis and bovine tuberculosis. However, one limitation of this study is the relatively small number of well-characterized *M. bovis* positive serum samples available for assay validation. Therefore, further studies involving larger sample sizes, especially more *M. bovis* positive clinical samples, are required to comprehensively evaluate the diagnostic performance of the developed test strip.

## 5 Conclusion

In conclusion, a fluorescent microsphere-based dual-antibody rapid detection method was successfully established for the simultaneous detection of antibodies against *Brucella* spp. and *M. bovis*. The developed method demonstrated high sensitivity and specificity, reduced testing costs, and improved detection efficiency. These findings suggest that this test strip may serve as a practical tool for rapid on-site screening of bovine brucellosis and bovine tuberculosis in field and primary-level settings.

## Data Availability

The original contributions presented in the study are included in the article/supplementary material, further inquiries can be directed to the corresponding authors.
